# Diversity of Rickettsia in Ticks Collected in Abruzzi and Molise Regions (Central Italy)

**DOI:** 10.3390/microorganisms7120696

**Published:** 2019-12-13

**Authors:** Ilaria Pascucci, Marco Di Domenico, Valentina Curini, Antonio Cocco, Daniela Averaimo, Nicola D’Alterio, Cesare Cammà

**Affiliations:** Istituto Zooprofilattico Sperimentale dell’Abruzzo e del Molise “G. Caporale”, 64100 Teramo, Italy; m.didomenico@izs.it (M.D.D.); v.curini@izs.it (V.C.); a.cocco@izs.it (A.C.); d.averaimo@izs.it (D.A.); n.dalterio@izs.it (N.D.); c.camma@izs.it (C.C.)

**Keywords:** ticks, SFG *Rickettsiae*, host/parasite relationship, biodiversity, wildlife

## Abstract

*Rickettsiae* have worldwide occurrence and rickettsiosis are widely recognized as emerging infections in several parts of the world. For decades, it was thought that a single pathogenic tick-borne spotted fever group (SFG), Rickettsia, occurred in each continent. Nowadays, thanks to molecular biology, new species of Rickettsia responsible for disease in humans are continuously identified worldwide. In a framework of diagnostic activities of the Istituto Zooprofilattico Sperimentale dell’Abruzzo e del Molise “G. Gaporale” and considering some reports of suspected human clinical cases of rickettsiosis, a survey on ticks collected form animals and humans was carried out with the aim to identify the *Rickettsia* species circulating in Abruzzi and Molise regions. A total of 603 ticks, previously identified at species level by morphology, pooled into 178 tick samples, were tested by pan-*Rickettsia* RealTime PCR. DNA from specimens positive for *Rickettsia* spp. was then sequenced in order to identify the *Rickettsia* species involved. The highest infection rate was detected in *Dermacentor marginatus* followed by *Ixodes ricinus*. The selected targets for this purpose were *OmpA* and *gltA. Rickettsia slovaca, Rickettsia monacensis, Rickettsia massiliae, Rickettsia conorii, Rickettsia aeschlimannii, Rickettsia helvetica, Rickettsia raoultii,* and *Rickettsia felis* – like organisms were identified in this study. These are the first data available in the literature for the circulation of SFG *Rickettsia* species in the selected geographical area. Results made evidence of high rate of infection in ticks. All *Rickettsia* species detected have been previously involved in human infection. The diversity of *Rickettsia* detected, and tick species collected reflects the biodiversity in term of wildlife and environment of the area. An association between *Rickettsia* species, ticks, and the relationships with vertebrate host species are discussed. Due to the peculiar eco-biology of each *Rickettsia* species, the use of diagnostic tools able to identify *Rickettsia* at the species level is thus recommended in order to assess the risk for humans and to elucidate more precise etiological diagnosis in clinical cases.

## 1. Introduction

*Rickettsiae* (family Rickettsiaceae, order Rickettsiales) are obligate intracellular bacteria transmitted by ticks, fleas, lice, and mites. Members of the genus *Rickettsia* can be classified into the spotted fever group (SFG) *Rickettsiae*, typhus group *Rickettsiae*, the *Rickettsia bellii* group, and the *Rickettsia canadensis* group [1]. *Rickettsiae* have worldwide occurrence and rickettsiosis are widely recognized as emerging infections in several parts of the globe, amongst these, spotted fever has a prominent role (2). For decades, it was thought that a single pathogenic tick-borne SFG *Rickettsia* occurred in each continent. Moreover, different *Rickettsia* species isolated from ticks were not considered pathogenic for humans for years or decades until a definitive association with human disease was established, such as the emblematic case of *Rickettsia massiliae* in Europe [2]. Hard ticks are not only the main vectors of SFG *Rickettsiae*, but also their main reservoir, even if an amplifier role of vertebrate species is suspected or to be clarified for some SFG *Rickettsia* species [1]. Vertical transmission and mainly an effective transovaric transmission (TOT) with a high level of filial infection rate (FIR), as described in ticks for the associated Rickettsia species, are the main ways of maintaining an infection in tick populations [1,3,4]. For example, for *R. massiliae* in *Rhipicephalus turanicus* ticks, a TOT of 100% and FIR of 98.5% have been reported [5]. For *Rickettsia africae* a TOT 100% and a FIR of 93.4% was also described in *Amblyomma variegatum* ticks [4]. *Rickettsia conorii* subspecies (sbsp) *conorii* the agent of Mediterranean spotted fever (MSF) is considered endemic mainly in the central and southern Regions of the Mediterranean [2]. Countries of the Mediterranean basin have noticed an increased incidence of MSF over the past 10 years. In Portugal, an annual incidence rate of 9.8 cases per 100,000 persons, the highest of the rates of all Mediterranean countries, has been reported [6,7]. In Italy, according to the Italian National Institute of Health [8], around 10,000 cases have been reported in the period 1996–2009. These cases are mainly described in warmer areas of the country, namely in central southern Italy and in the main islands. Four regions, Sicily, Sardinia, Latium, and Calabria contained more the 85% of total cases, generally reported during the long hot season from July to October, thus according with the seasonal dynamic of the main vector *Rhipicepahlus sanguineus* that behaves as an exophilic tick at those latitudes [8,9]. More recently, an average incidence of 21.17–176.88 cases per million persons of Rickettsiosis has been reported from 2009 to 2013 [10]. This rate likely underestimates the true incidence, and some authors suggest that there are seven times more cases than officially reported [6] since a great part of the diagnosis is made by clinical symptoms and serology without the identification of the pathogen involved. For many years, it was assumed that *R. conorii* sbsp *conorii* was the only agent of SFG *Rickettsia* circulating in Italy, and that the classical clinical manifestation of MSF was the only one developed after the infection in humans. In the last decades, thanks to the improved diagnostic skills and, mainly, to the use of molecular tools, different SFG *Rickettsia* have been identified in the Italian territory including *Rickettsia slovaca, Rickettsia aeschlimanni, Rickettsia massilliae, Rickettsia monacensis, Rickettsia conorii* subspecies*. Israelensis, Rickettsia conorii* subsp. *Indica, Rickettsia raoulti, Rickettsia helvetica,* and *Rickettsia hoogstraalii,* [1,2,11,12,13,14].

In Abruzzi and Molise according the Ministry of Health in the period from 1996–2009, 81 and 43 human cases respectively, were notified. However, the presence of subclinical forms that may be undetected and the possible circulation of *Rickettsia* species that can cause symptoms different from the classical MSF could have led to an underreporting. Moreover, cases of hospitalized human Rickettsiosis have been reported during the time 2001–2015 [8,15] and human cases are still presently diagnosed at the local hospitals. Likewise, previous preliminary studies on tick borne diseases carried out in the area showed the circulation of different *Rickettsi*a species such as *R. slovaca* [16] and *R. monacensis* [17]. The present study aims to investigate the possible circulation of *Rickettsia* species, and to identify species of interest for human health in Abruzzi and Molise Regions. 

## 2. Materials and Methods 

The Istituto Zooprofilattico Sperimentale dell’Abruzzo e del Molise “G. Caporale” is a public health institute which operates in the Abruzzi and Molise Regions, performing analytical work for public veterinary services, and providing the technical and scientific collaboration necessary to enable them to carry out their functions in the field of veterinary public health. The diagnostic department receives carcasses of domestic and wild animals for diagnostic purposes and in the framework of specific surveillance and monitoring programs such as the one on wildlife health status. Working under the same ministry, it receives samples from hospitals as well in cases of suspected infection with a zoonotic agent. Considering reports of suspected human clinical cases of Rickettsiosis and a lack of detailed information for the area, a passive survey on *Rickettsia* in ticks collected from domestic and wild animals and humans received at the diagnostic department from 2014 to 2106 was carried out.

### 2.1. Study Area

With an altitude from sea level to over 2900 m. above sea level. the study area covers different environments and it is characterized by high biodiversity in terms wildlife with 36% of the territory protected in three national parks and many natural reserves. At least 2 endemic endangered mammals species are present in the region, such as the Marsican brown bear (*Ursus arctos marsicanus*) and Apennine chamois (*Rupicapra pyrenaica ornata*), but the environmental features of the area are able to support a high density of wild ungulates (roe deer—*Capreolus capreolus*, red deer—*Cervus elaphus,* and wild boar—*Sus scrofa*) and wild carnivorous (wolf—*Canis lupus*, European badger—*Meles meles*, and red fox—*Vulpes vulpes*). Meanwhile, rural farming is common in the area, especially for small ruminants. 

### 2.2. Tick Collection and Identification

From January 2014 to the end of December 2016, ticks were collected during post mortem examination, placed in a vial, and identified with an identification number. In the case of ticks collected from animals, the ID number was the same as the carcass to which they belong. Collection data (host species, date, municipality) were registered in the laboratory informative system during the acceptance of the carcass or tick. Ticks were preserved in 70% ethanol and subsequently identified at the species level and developmental stage by morphology using the dichotomous key described by Manilla [9].

### 2.3. Rickettsia DNA Detection and Identification

DNA from all from samples (pools) was extracted by the Maxwell 16 Tissue DNA Purification Kit (Promega) and analyzed by pan-Rickettsia Real-Time PCR [18] targeting 23S rRna gene. The reaction mixture consisted of 20 µL containing 2X GoTaq Probe qPCR Master Mix (Promega, Madison, US), 250 nM of TaqMan probe (PanR8_P), 600 nM of each primer (PanR8_F and PanR8_R), 5 µL DNA and nuclease-free water up to final volume. The reaction protocol was used in fast mode: 20 s at 95 °C followed by 40 cycles of 1 s at 95 °C and 20 s at 60°C. The reaction was performed on the 7900HT Fast RealTime PCR System (Applied Biosystems, Foster City, US) and analysed by the sequence detection system software (SDS) v2.4 (Applied Biosystems). The RealTime PCR assay was always performed including a plasmid containing a portion of the Rickettsia 23S rRNA gene as positive control and No Template Control (NTC). *Rickettsia spp.* positive samples were further investigated by sequencing a portion of OmpA gene and gltA gene [19]. Five µl of purified DNA were amplified in a 50 µLreaction mixture containing 300 nM of each forward and reverse primer, 200 µM dNTPs (Promega), 2.5 mM MgCl_2_ Solution (Applied Biosystem), 0.125 U/µL AmpliTaq Gold (Applied Biosystem) and 1 × PCR Buffer II (Applied Biosystem). Amplifications were carried out in a GeneAmp PCR System 9700 (Applied Biosystems) under the following conditions: initial denaturation 10 min at 94 °C, followed by 45 cycles of denaturation (30 s at 94 °C), annealing (30 s at 56 °C), and extension (30 s at 72 °C). The final extension was completed at 72 °C for 7 min. PCR products were purified using the GeneAll Expin PCR Kit (GeneAll, Seoul, Korea) and sequenced by the BigDye Terminator v.3.1 (Applied Biosystems). Analyses were performed on the 3130 XL Genetic Analyzer (Applied Biosystems). Raw sequence data were assembled using Vector NTI suite 9.1 (Invitrogen, Waltham, US) 

## 3. Results 

**Figure 1:**
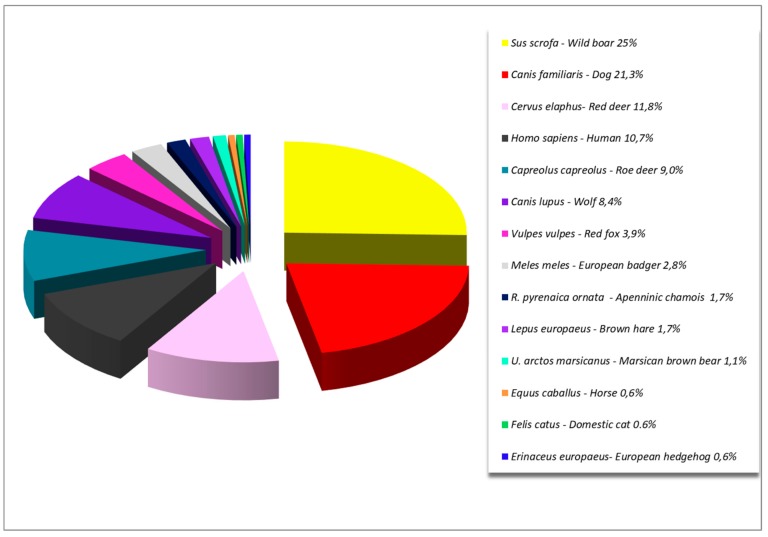
Distribution of tick-pools according host species.

Ticks were collected from 15 different host species including humans while 584 ticks from different wild and domestic species and 19 ticks biting humans were included, for a total of 603 adult ticks. Pools were created for each single host, according tick species and developmental stage. Totally 178 adult tick pools of 1–5 individuals each, were tested. Wild boars (*Sus scrofa*) followed by cervids (*Capreolus capreolus* and *Cervus elaphus*) and dogs (*Canis familiaris*) were the greatest contributors to the collection (Figure 1).

Tick species: seven tick species were detected and the most frequent in the collection was *I. Ricinus,* followed by *D. marginatus* and *R. sanguineus* sensu lato. In Table 1, results of the identification of ticks are shown. The most common tick on humans was *I. ricinus,* but *R. turanicus*, *D. marginatus*, and *Ixodes hexagonus* were also detected. 

Infection with *Rickettsia* spp.: the percentage of positivity for *Rickettsia* spp. was expressed as minimum infection rate (MIR) assuming that one tick was positive in each positive pool. The highest MIR was detected in *D. marginatus* (81.1%) followed by *I. ricinus* (53.5%).

**Figure 2:**
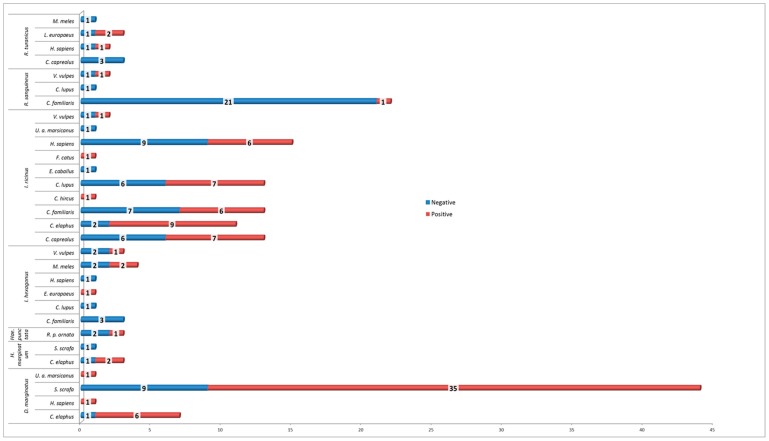
Details of RealTime *Rickettsia* spp. positive tick-pools/host association.

Positive tick–host association: the most important contribution of *D. marginatus* positive tick pools was provided by the wild boar (35/43) with a lesser extent by red deer (6/43), while for *I. ricinus* positive pools were more homogeneously distributed amongst the different hosts including humans. Eight out of the 19 ticks collected from human beings, tested positive (42%). Figure 2 shows the details of RealTime *Rickettsia* spp. positive tick–host association.

*Rickettsia*–tick species association: eight species of *Rickettsia* have been detected in 93 positive pools. *R. slovaca* and *R. monacensis* were 85% of the positives. In term of ticks, the only species found infected with *R. slovaca* was *D. marginatus,* while *R. monacensis* was mainly identified in *I. ricinus* with one positive pool of *D. marginatus, R. sanguineus* and *Haemaphysalis punctata* respectively. 

*Rickettsia*–tick species-host association: in term of vertebrate host, *D. marginatus* ticks infected by *R. slovaca* were mainly carried by wild boar (*S. scrofa*) in 34 of 41 cases, with the contribution of red deer (*C. elaphus*) at 6 in 34, and of a sample from humans, while a wider host range including five samples from humans contributed to the amount of *I. ricinus* infected with *R. monacensis*, which was the most frequent species detected in ticks collected from humans (5 out of 19), where *R. massiliae* and *R. slovaca* were also identified. 

In Table 2, data on *Rickettsia* species in relation to ticks and host species are shown.

## 4. Discussion

To our knowledge, these are the first comprehensive data that investigate *Rickettsia* circulation in the environment in the study area and even if significant bias affected the passive feature of the monitoring (mainly wild host and according seasonality and hunting), they provide important information to assess the possible risk for humans. Results demonstrated evidence of a high rate of infection (52.2%) for *Rickettsia* spp in ticks (93 positive pools out of 178). The infection rate was higher than what has been reported in a similar study carried out on 77 pools of three ticks by Ebani and collaborators [20] in Tuscany (20.78%) and higher than what has been reported by Scarpulla and collaborators [12] on 113 ticks collected from host and environment in Latium and Tuscany regions (12.4%) and tested singularly (38), or in a pool of 10 ticks (18). Likewise, our infection rate was higher than what has been recently reported in a study carried out testing free living ticks in a sub urban area in Latium 25.8% where however 255 ticks had been selected randomly out of 518 ticks, and singularly tested [21]. The wide range of tick species collected reflects the biodiversity in term of wildlife and environments. *I. ricinus* has been confirmed as a generalist ticks with the broadest host range (10 species out of 15) and as the most frequent on humans (15 out of 19) confirming the literature data reporting several hundred species of animals included in the host range [9,22]. Another *Ixodes* tick frequent in our collection was *I. hexagonus*, a species associated with the European hedgehog (*Erinaceus europeus)* and its predators [9,23] that seldom bites humans. As a confirmation of the literature, it has been found in our survey in hedgehog and in different carnivorous species, such as the European badger (*Meles meles*), red fox (*Vulpes vulpes*), wolf (*Canis lupus*), and domestic dogs. The ticks *R. sanguigneus* has been found mainly on dogs as known in literature [9], but also on other canids. Samples of this ticks were not further investigated in order to identify different lineages that, according Zemtsova and collaborators [24], are circulating in the temperate zone. The closely related species *R. turanicus* was also collected from a wider host-range, including human. Furthermore, our findings confirm the strict association between wild boar and *D. marginatus* just as described in other studies in Southern Europe. A strong host–parasite association was observed between wild boar and adult *D. marginatus* by Selmi and collaborators in a study performed in the North West Mediterranean region [25] wherein 99.7% of adult *D. marginatus* ticks obtained from wild ungulates were collected on wild boars. Similarly, in our study, 83% of *D. marginatus* ticks were found on wild boar. Selmi and collaborators [25] hypothesize that wild boar plays a role in *D. marginatus* breeding aggregations. The only other species found on wild boar in our survey was *Hyalomma marginatum,* a xerophilic tick that usually as adult feeds on large ungulates such as bovine and equids but also on red deer and seldom on wild boar [9]. The small number of *H. marginatum* likely depends to both, the scarcity in our collection of big ungulates and the environmental features of the collection areas (mainly humid biotopes). Furthermore, the detection of *Hae. punctata* on the endemic species, Apennine chamois, is a common finding in the area. The reason for this association between the wild ungulate and tick species is probably to be sought in the sharing of habitat type while both, in fact, are commonly found in high rocky pastures and not in the forested area of the Mediterranean [9]. 

In our study, eight SFG Rickettsia species have been identified in ticks: *R. slovaca*, *R. monacensis*, *R. massiliae*, *R. conorii*, *R. aeschlimannii*, *R. helvetica*, *R. raoultii*, and *R. felis*. The findings on *Rickettsia* species of our study not completely overlap with the findings of similar studies carried out by Scarpulla and collaborators [12] in Tuscany, Maioli and collaborators [11] in Emilia Romagna, and Chisu and collaborators [26] in Sardinia. *R. monacensis* the second most representative species in our study was the most frequent species in Tuscany [12], and previously in Emilia Romagna [11], while it was not detected in Sardinia [26]. The reasons for the difference are mainly found in the tick composition of the collection that is affected by the host, and also by the environmental features of the study area, that indirectly affect the *Rickettsia* species composition*. R. monacensis* and *R. helvetica* in fact are both mainly transmitted by *I. ricinus* that is scarcely present in Sardinia [27]. In our study, *R. helvetica* was identified exclusively in *I. ricinus* pools (two) which are the main vector and natural reservoir [1], while *R. monacensis* was found mainly in *I. ricinus* pools (35 out of 38), with one positive in *D. marginatus*, *R. sanguineus* and *Hae.punctata*—the latter is similar to the finding of Scarpulla and collaborators [12]. These findings suggest that, even if the circulation in the environment is mainly maintained by *I. ricinus*, there may be other species implicated in the transmission or *R. monacensis* as suggested by Madeddu and collaborators [28]. Both *Rickettsia* species have been implicated in human cases of spotted fever in Italy [2,28]. 

The most frequent *Rickettsia* identified was *R. slovaca* (44.1%—41 out of 93), with *R. raoultii* also detected alone and in co-infection in our study. *R. slovaca* is associated in humans with a syndrome characterized by scalp eschars and neck lymphadenopathy following tick bite. Initially, this syndrome was named TIBOLA (tick borne lymphadenopathy) or DEBONEL (*Dermacentor*-borne necrotic erythema and lymphadenopathy) and after 2010, SENLAT (scalp eschar and neck lymphadenopathy after a tick bite) [1] because of its strict but not exclusive association with *Dermacentor* ticks described also in our study. The positive percentage of *R. slovaca* in *D. marginatus* pools is notably high (79.5 %) compared with a similar study carried out in Emilia Romagna testing ticks singularly [11] where it was 22.7%. *Dermancento*r positive ticks were mainly collected from hunted wild boars (35 out of 53), however, positive results were also obtained in ticks from red deer and humans. Our results, as well as the data reported in the literature, [11,13] suggest a possible role of the wild boar in the transmission of these *Rickettsia*, but, according to different authors this remains to be demonstrated. To support the latter statement, a preliminary survey was previously performed in L’Aquila province to assess the presence of emerging tick-borne bacteria in wild boar, testing by RealTime PCR for *Rickettsia* spp., *Francisella tularensis, Coxiella burnetii, Anaplasma phagocytophilum* ticks, and spleen samples of hunted wild boars. In this study, out of 33 pools of *D. marginatus,* 27 tested positive for *Rickettsia slovaca.* Conversely, none of the 104 spleen samples gave positive results for *Rickettsia* spp. [16]. Considering that in Italy, as for all around Europe, an increased density of wild boars has been reported during the past few decades [29,30], and that hunting pressure probably induced an adaptive behavior of the wild boar, making it able to live in close proximity to human activities [25] and enhancing the risk of contact between humans and *R. slovaca* [13], the level of awareness of this pathogen should be raised.

Furthermore, SFG *Rickettsiae*, long ago recognized as agents of human diseases also in Italy [2], were detected in our study such as *R. conorii* and *R. massiliae* both only in *Rhipicephalus* ticks confirming literature data that describe *R. massiliae* presence all over *R. turanicus* and *R. sanguineus* geographic distribution [1]. Our results corroborate moreover the strict vector/ pathogen association, being the two *Rickettsiae* detected only in *Rhipicephalus* ticks: *R. conorii* only in *R. turanicus,* while *R. massiliae* in both *Rhipicepahlus* tick species. It is noteworthy that out of nine pools of *R. turanicus* tested, two were positive for *R. massiliae.* This is not surprising considering that a study on naturally infected ticks showed 98.5% FIR of *Rickettsia massiliae* in *R. turanicus* ticks [5]. Similarly, the relatively low general MIR for *R. conorii* (0.6%) and its absence in *R. sanguineus* is in accordance with experimental results and field data that demonstrated the excellent fitness of a *R. conorii*-infected tick laboratory colony that diverges from the low prevalence of infected *R. sanguineus* ticks collected in the wild (–1%), and thus even in endemic areas [1,31]. These observations suggest a possible involvement of undetermined vertebrate host in the life cycle of *R. conorii.* The detection of *R. raoultii* confirms literature data. *R. raoultii* has just been implicated in cases of DEBONEL/TIBOLA [32]. In Italy it has just been reported in Liguria in *D. marginatus* ticks [13] and in Sardinia in *R. sanguineus* and *D. marginatus* ticks. *R. raoultii* is mainly associated with both European *Dermacentor* ticks, *D. marginatus* and *D. reticulatus* that are able to transmit it to the progeny. However, *R. raoultii* prevalence in ticks is extremely variable according to the predominant species in the area and the concomitant circulation of *R. slovaca*. Generally, *R. raoultii* is mainly associated to *D. reticulatus*, absent in a great part of Italian territory, and where this tick is not present it usually circulates in *D. marginatus* with a lower infection rate than *R. slovaca* [13]. The latter condition will suit our findings.

Our results on *R. aeschilmanni* are in line with literature data that describe *R. aeschilmanni* in *Hyalomma* ticks as a common finding all around Southern Europe [1], including Italy [ 12]. Literature data suggest that this *Rickettsia* may be spread through migratory birds carrying immature *Hylomma rufipes* ticks from Africa or *Hylomma marginatum* through the Mediterranean [33,34,35]. Since its detection in Morocco in 1997, *H. marginatum* [36] has indeed been implicated in human spotted fever, demonstrating a primary role among the emerging pathogens also in our country [37]. 

The detection of *R. felis* in *I. hexagonus* ticks is an original finding, but it needs further investigation: *R. felis* is commonly recognized as an emergent pathogen common cause of febrile illness in humans, mainly in sub-Saharan Africa. Nevertheless, its taxonomy is tricky and the mechanism of genetic evolution remains under study. At the beginning, according to an *ompA* gene sequence, it was included in the SFG. Afterwards, more detailed phylogenetic analysis placed *R. felis* together with *Rickettsia akari* and *R. hoogstraalii* in a separate ‘transitional’ group that has features of both SFG and the typhus group (TG) of *Rickettsiae* [38], the identification of the bacteria belonging to this transitional group is still ongoing. According to Angelakis and collaborators [38], a great part of the worldwide reports of ‘*R. felis*’ are probably not given by the type strain of *R. felis*, but rather by other genetically related species. All of these strains or genetic variants were reported as *R. felis*, but they probably represent a not yet isolated species within the *R. felis* cluster for these defined *R. felis*-like organisms (RFLOs) that, in contrast to the classical *R. felis*, are widely distributed in arthropods and are not able to cause the disease in humans [38]. To date, it has been assigned to the cat flea (*Ctenocephalides felis*), the role of the only confirmed biological vector of *R. felis*, while RFLOs have been identified in more than 20 different hematophagous species of flea, mosquito, as well as soft and hard ticks all around the world [38].

In Europe, RFLO infection was initially detected in *Hae. sulcata* in Croatia [39], but successively identified as *R. hoogstraalii* infection [40], and recently in southern Italy [41] in *R. turanicus*.

Conversely, in our study, RFLOs were only detected in *I. hexagonus.* The reason for this association may be found in the host preference of ticks, but further investigations are needed considering that *R. felis* strains isolated from different arthropods shows host-specific genomic variation [42].

The detection of *R. monacensis*, *R. helvetica*, *R. massiliae,* and *R. slovaca* in the 19 samples from humans is in accordance with the above description for tick–*Rickettsia* species association. Accordingly, public awareness should be increased, demonstrating how easy it is for humans in the area to come in contact with these pathogens.

The wide range of tick species collected reflects the biodiversity in terms of wildlife and environments of the area, and subsequently the variety of tick species directly affects the diversity of *Rickettsia* species identified in our survey. *Rickettsiae* have, in fact, developed many strategies to adapt to different environmental conditions, including those within their arthropod vectors and vertebrate hosts [3]. Due to the peculiar bio-ecological features of each *Rickettsia* species detected, the use of diagnostic tools able to identify *Rickettsia* at the species level, it is recommended to assess the risk for humans and to elucidate more precise etiological diagnosis in clinical cases

According Merhej and collaborators [43], molecular studies have revolutionized the systematic study of *Rickettsia* and unveiled the great diversity of *Rickettsia* spp. with respect to host ranges, effects on hosts, and geographical distribution. 

## 5. Conclusions

Our results are the first comprehensive data available for *Rickettsia* spp. circulation in the area and can be considered a baseline study to assess the possible risk for humans. This type of information is even more necessary in geographical areas, such as the study area here, characterized by human activities that take place in a rural context in a conserved natural environment rich in wildlife, wherein humans, domestic and wild animals, vectors, and transmitted pathogens interact easily.

## Figures and Tables

**Table 1:** details of the results of RealTime PCR for *Rickettsia* spp. in each tick species.

Table	Negative	Positive	Total	Tick Species Frequency	Minimum Infection Rate
*I. ricinus*	33	38	71	39.9%	53.5%
*D. marginatus*	10	43	53	29.8%	81.1%
*R. sanguineus*	23	2	25	14.0%	8.0%
*I. hexagonus*	9	4	13	7.3%	30.8%
*R. turanicus*	6	3	9	5.1%	33.3%
*H. marginatum*	2	2	4	2.2%	50.0%
*Hae. punctata*	2	1	3	1.7%	33.3%
Total	85	93	178		52.25%

**Table 2:** Rickettsia species in relation to tick and host species.

*Rickettsia* species	Host	Ticks									
		*D. marginatus*	*H. marginatum*	*Hae. punctata*	*I. hexagonus*	*I. ricinus*	*R. sanguineus s.l.*	*R. turanicus*	Total	%	*Total MIR*
*R. conorii*	*L. europaeus*	/	/	/	/	/	/	1	/	/	/
*R. conorii* Total		/	/	/	/	/	/	1	1	1.1%	0.6%
*R. felis*	*E. europaeus*	/	/	/	1	/	/	/	/	/	/
	*V. vulpes*	/	/	/	1	/	/	/	/	/	/
*R. felis* Total		/	/	/	2	/	/	/	2	2.2%	1.1%
*R. haeschlimanni*	*C. elaphus*	/	2	/	/	/	/	/	/	/	/
*R. haeschlimanni* Total	/	/	2	/	/	/	/	/	2	2.2%	1.1%
*R.helvetica*	*H. sapiens*	/	/	/	/	1	/	/	/	/	/
	*V. vulpes*	/	/	/	/	1	/	/	/	/	/
*R. helvetica Total*	/	/	/	/	/	2	/	/	2	2.2%	1.1%
*R. massiliae*	*H. sapiens*	/	/	/	/	/	/	1	/	/	/
	*L. europaeus*	/	/	/	/	/	/	1	/	/	/
	*V. vulpes*	/	/	/	/	/	1	/	/	/	/
*R. massiliae* Total	/	/	/	/	/	/	1	2	3	3.2%	1.7%
*R. monacensis*	*C. capreolus*	/	/	/	/	7	/	/	/	/	/
	*C. elaphus*	/	/	/	/	8	/	/	/	/	/
	*C. familiaris*	/	/	/	/	6	1	/	/	/	/
	*C. lupus*	/	/	/	/	7	/	/	/	/	/
	*F. catus*	/	/	/	/	1	/	/	/	/	/
	*H. sapiens*	/	/	/	/	5	/	/	/	/	/
	*R. p. ornata*	/	/	1	/	/	/	/	/	/	/
	*U. a. marsicanus*	1	/	/	/	/	/	/	/	/	/
	*C. hircus*	/	/	/	/	1	/	/	/	/	/
*R. monacensis* Total	/	1	/	1	/	35	1	/	38	40.9%	21.3%
*R. raoultii/R. slovaca*	*C. elaphus*	/	/	/	/	1	/	/	/	/	/
	*M. meles*	/	/	/	1	/	/	/	/	/	/
	*S. scrofa*	1	/	/	/	/	/	/	/	/	/
*R. raoultii/R. slovaca* Total	/	1	/	/	1	1	/	/	3	3.2%	1.7%
*R. slovaca*	*C. elaphus*	6	/	/	/	/	/	/	/	/	/
	*H. sapiens*	1	/	/	/	/	/	/	/	/	/
	*S. scrofa*	34	/	/	/	/	/	/	/	/	/
*R. slovaca* Total	/	41	/	/	/	/	/	/	41	44.1%	23.0%
*R. raoultii*	*M. meles*	/	/	/	1	/	/	/	/	/	/
*R. raoultii* Total	/		/	/	1	/	/	/	1	1,1%	0.6%
Total	/	43	2	1	4	38	2	3	93	100%	52.2%

Quantitative data on Rickettsia species are shown in relation to tick and host species.
